# 747 Standardization of Upper Extremity Elevation

**DOI:** 10.1093/jbcr/irac012.300

**Published:** 2022-03-23

**Authors:** Hannah Miller

**Affiliations:** The University of Kansas Health System, Blue Springs, Missouri

## Abstract

**Introduction:**

Elevation of burn-injured extremities is crucial to preserve function, prevent contracture and scar formation. Limited data leading to various techniques utilized across burn centers has resulted in no local, national, or international standard of practice. The absence of standardized documentation of these interventions prevents data tracking and outcome analysis.

**Methods:**

A literature review was conducted to assess the various methods of upper extremity elevation in burn patients. A request was sent to ABA verified burn centers to collect feedback on elevation practices. The interdisciplinary team was consulted for their professional expertise and opinion. The unit practice council reviewed all data to establish a standard of practice for upper extremity elevation. A procedure was written explaining the purpose, proper implementation, and potential complications. Documentation in the electronic medical record (EMR) was updated to reflect the practice change. Education will be disseminated to bedside staff. Return demonstration will be required, completed with a trained validator to ensure staff competency. Data will be collected and analyzed through EMR audits.

**Results:**

Information gathered via literature review proves there is inconsistent practice of upper extremity elevation post-burn injury. ABA verified burn center survey results support the current literature findings, and the importance elevation plays in preserving function and quality of life in burn survivors.

**Conclusions:**

Upper extremities are frequently impacted by burn injury, potentially resulting in significant disability. A common physical complication of burn injury is contracture of major joints, leading to further surgical intervention and/or permanent disability. Standardizing the practice of upper extremity elevation has the potential to preserve joint function and range-of-motion. A procedure has been written and published hospital-wide. Staff compliance and documentation audits will assist in evaluating the efficacy of the upper extremity elevation. Barriers to optimal outcomes include staff compliance, documentation inconsistencies, and limited sample size.

